# Septic Arthritis Caused by Sphingomonas Paucimobilis in Immunocompetent Patient: Case Report and Literature Review

**DOI:** 10.7759/cureus.22167

**Published:** 2022-02-13

**Authors:** Mohammed K Alageel, Sarah I Alromaih, Mohammed T Alzahrani, Adham M Abdulsamad

**Affiliations:** 1 Orthopedics, College of Medicine, King Saud bin Abdulaziz University for Health Sciences, Riyadh, SAU; 2 Department of Surgery, Division of Orthopedic Surgery, King Abdulaziz Medical City, Ministry of National Guard Health Affairs, Riyadh, SAU

**Keywords:** monoarthritis, case report, immunocompetent, sphingomonas paucimobilis, septic arthritis

## Abstract

Septic arthritis, also known as infectious arthritis, is inflammation of the joints due to a wide range of pathogens, such as bacterial, fungal, mycobacterial, viral, or/and other pathogens; however, some opportunistic pathogens tend to affect immunocompromised patients and rarely infect immunocompetent patients. For example, *Sphingomonas* *paucimobilis* is an opportunistic pathogen with a particular tropism toward bones and soft tissues that rarely causes infections in immunocompetent humans. We present a case of *Sphingomonas paucimobilis* causing septic arthritis in a 34-year-old man who is medically free and with no history of previous surgeries or any other comorbidities. He was treated successfully by both pharmacological treatment and surgical intervention. To our knowledge, there are only four cases published in the literature involving *Sphingomonas paucimobilis* as a causative organism of septic arthritis affecting immunocompetent patients.

## Introduction

Septic arthritis is an inflammation of the joint due to a wide range of pathogens and characteristically results in significant rates of morbidity and mortality [1]. According to an England and Wales study, 68.6% of the cases of septic arthritis were caused by staphylococcal and streptococcal species [2]. Similarly, a study conducted in Riyadh, Saudi Arabia, found staphylococcal and streptococcal species caused more than 75% of septic arthritis cases [3]. However, *Sphingomonas paucimobilis*, Gram-negative yellow-pigmented non-fermenting bacilli, is an opportunistic pathogen with a particular tropism toward bones and soft tissues that rarely causes infections in immunocompetent humans [4]. To our knowledge, there are only four cases published in the literature involving *Sphingomonas paucimobilis* as a causative organism of septic arthritis affecting immunocompetent patients. Our aim is to report an uncommon septic arthritis case caused by an opportunistic infection and review the previously published cases.

## Case presentation

We present a case of a 34-year-old man without any comorbidity or previous medical conditions and with no history of previous surgeries. The patient is a smoker, and he denied traveling or other risky behaviors. He had low energy non-penetrating trauma to his right knee, and he was able to ambulate immediately. When he heard a cracking sound and felt pain, he went directly to the emergency department complaining of knee pain, swelling, and effusion. Physical examination revealed effusion with an inability to fully raise his leg with only 15 degrees of leg extension lag. Suspicion of quadriceps or patellar tendon injury was ruled out by knee ultrasound which showed only effusion. Moreover, examination of the knee revealed a stable knee, range of motion 15-100 degrees, with intact medial and lateral collateral ligaments. Due to pain, further ligamentous examination for the anterior cruciate ligament and posterior cruciate ligament was not conducted. The patient was referred to outpatient for revaluation after resolution of effusion and possible MRI. After two weeks, he attended the outpatient clinic where he complained of progressing knee effusion and pain. The pain was sudden, continuous, diffuse with no relieving and aggravating factors. The pain was severe to the extent that sometimes he could not bear weight on his right knee. On physical examination, he was afebrile and hemodynamically stable. His right knee was swollen, warm, tender, with no open wounds, limited active and passive range of motion from 15-40 degrees due to pain.

In the emergency department, the right knee X-ray showed no definite fracture or dislocation; however, it showed mild suprapatellar effusion. In the clinic, we decided to aspirate his right knee. The aspiration showed cloudy yellow turbid synovial fluid. The microbiological studies confirmed the presence of gram-negative bacilli, and the result verified pan-sensitive *Sphingomonas paucimobilis* infection after four days when the patient was immediately admitted from the clinic. Upon the first outpatient clinic visit, which was after two weeks, the blood investigations were as follows: white blood cells, 4.3×10^9^/L; hemoglobin, 126 g/L; platelets, 440×10^9^/L; C-reactive protein, 102 mg/L; erythrocyte sedimentation rate, 81 mm/h. However, after 4 days, on admission, they were as follows: white blood cells, 5.7×10^9^/L; hemoglobin, 121 g/L; platelets, 448×10^9^/L; C-reactive protein, 75 mg/L; erythrocyte sedimentation rate, 57 mm/h. 

Upon admission, as a standardized hospital reference, the infectious disease team was consulted and started on 2g intravenous ceftazidime every 8 hours (q8h). On the following day, the patient underwent arthroscopic irrigation and debridement of the right knee, and the pathology result revealed the presence of acute inflammatory cells and fibrin which is consistent with septic arthritis. After the procedure, he was on a small Jackson-Pratt drain (Cardinal Health, Ohio, USA), which drained less than 100 cc of hemoserous fluid for the three postoperative days. On the second postoperative day, the infectious disease team recommends adding a loading dose of vancomycin 1300 mg simultaneously with ceftazidime, followed by regular vancomycin dose 1g q8h. However, the patient developed an allergic reaction. Thus the vancomycin was replaced with linezolid 600 mg every 12 hours (q12H). The patient improved and was discharged on the eighth postoperative day to continue on oral linezolid 600 mg q12H for four weeks and ciprofloxacin 500 mg q12H both for four weeks; however, unfortunately, the patient did not start the oral antibiotics until the next outpatient visit that was 11 days after the discharge. Despite the clinical improvement in pain and knee range of motion, He was instructed to start the antibiotics immediately, and he was given a follow-up after one week. After four weeks of antibiotics course, he was seen in the clinic doing fine and had a full recovery.

## Discussion

*Sphingomonas paucimobilis* has been reported as one of the causative organisms of nosocomial and community-acquired infections associated with different comorbidities [5]. Moreover, it has a variable pattern of antibiotic susceptibility. It is reported to be susceptible to third-generation cephalosporins and fluoroquinolones and resistant to penicillin and first-generation cephalosporins [6]. 

Septic arthritis with *Sphingomonas paucimobilis *is a rare entity. The literature review has revealed only four cases regarding *Sphingomonas paucimobilis* causing septic arthritis in immunocompetent which are listed in Table 1.

**Table 1:** Published cases of Sphingomonas paucimobilis producing septic arthritis in immunocompetent patients Kuo et al. [7]; Souto et al. [8]; Dischereit et al. [9]; Pascale et al. [10]

Case	Age by years	Affected joint	Medical and surgical history	Surgical management	Time-delay of surgical intervention	Length of stay	Outcome	Follow-up
Kuo et al.	47	Right knee	The patient suffers from diabetes mellitus, gouty arthritis, and received vacuum cupping over the skin of his right knee just a few days before the presentation.	Yes	On the 12^th^ day of admission	Five weeks	Complete remission	six months
Souto et al.	41	Left knee	He is known to have hyperuricemia.	Yes	After 22 days of treatment	More than three weeks	Complete remission	Not mentioned
Dischereit et al.	70	Right knee	The patient has hypertension, hyperuricemia, chronic renal failure stage III, aortic valve replacement, and stage II-III COPD	No	Not applicable	Not mentioned	Infection was treated successfully with a considerable improvement of the symptoms	Not mentioned
Pascale et al.	26	Right knee	He underwent corrective surgery for limb length inequality two months ago for a tibia fracture.	No	Not applicable	Treated as an outpatient	Complete remission	Two months
Present case	34	Right knee	He is medically and surgically free.	Yes	Next day of admission	Nine days	Complete remission	Two months

In comparison to our study, the literature review showed our patient is considered unique since he is young and medically free, unlike what has been reported in the literature where all of them have musculoskeletal disorders such as gouty arthritis, hyperuricemia, and recent musculoskeletal surgery. Moreover, regarding the time of the surgery, we noticed that the early surgical intervention, which was the next day of admission for our patient, could be the reason for the shorter length of hospitalization.

In regard to medications, there was a wide range of diversity. Each case was susceptible to certain drugs and resistant to others. However, we found that third-generation cephalosporins and fluoroquinolones were frequently used and showed an improvement in patients' status. However, Pascale et al. used sulfonamides and amoxicillin/clavulanic acid when the susceptibility profile showed resistance to cephalosporins and fluoroquinolones [10]. According to the surgical intervention, the surgery was avoided in two cases, Pascale et al. [10] and Dischereit et al. [9]; the Dischereit et al. case was due to the risk of the surgery since the patient has a critical medical condition who suffer from chronic renal failure stage III, stage II-III chronic obstructive pulmonary disease (COPD), and the need to be on oral anticoagulation [9].

## Conclusions

Even though *Sphingomonas paucimobilis* is an opportunistic and nosocomial infection, it is not clear why and how it can affect immunocompetent patients. However, our patient had a full recovery after surgical and medical treatment. Further studies are needed to explain how it affects immunocompetent patients and the best treatment options.
